# Reconstruction of composite oral and maxillofacial defects by free flaps based on a new classification

**DOI:** 10.1038/s41598-020-61345-z

**Published:** 2020-03-09

**Authors:** Xi Yu Yao, Hui Liu, Wei Wei Liu

**Affiliations:** 1 Department of Head and Neck Surgery, Fujian Cancer Hospital & Fujian Medical University Cancer Hospital, Fuzhou, Fujian, 350011 P. R. China; 2 Department of Head and Neck Surgery, Key Laboratory of Oncology in Southern China, Cancer Center of SUN Yat-sen University, Guangzhou, 510060 P. R. China

**Keywords:** Cancer therapy, Head and neck cancer

## Abstract

Reconstruction of composite oral and maxillofacial defects using free flaps is challenging. The key to such delicate reconstruction lies in the evaluation of the defect. However, few reports have described the clinical classification of these difficult defects. In this study, we proposed a classification method and developed different reconstructive solutions using free flap techniques according to this classification. This classification method was established based on two parameters (the elements and distribution of the missing tissues). Among the 17 patients with composite oral and maxillofacial defects included in this study, 8 patients presented with type A defects, one patient presented with a type B defect, and the other 8 patients presented with type C defects. No type D defects were observed in this study. Three types of free flaps were used to reconstruct the respective types of composite defects. Perforator-based ALT flaps were used to reconstruct Type A defects. Branch-based ALT flaps were used to reconstruct Type B defects. For Type C defects, two reconstructive solutions were used, including a well-designed fibular flap and a perforator-based ALT flap with or without a metal plate for bony reconstruction. All flaps survived after surgery. This classification system may help select and design specific free flaps for reconstructing composite oral and maxillofacial defects.

## Introduction

Composite oral and maxillofacial defects after ablative oncological surgery, such as typical through-and-through oral defects, are challenging clinical problems for plastic and reconstructive surgeons. Multiple factors, including different related anatomical sites, multiple types of missing tissues, and sometimes even two separate defects, often cause these composite defects to be variable and complicated to reconstruct. Understanding the features or patterns of these composite defects may provide a framework to describe these defects and help plan the reconstructive algorithm. However, as we know, few studies have reported the classification of composite oral and maxillofacial defects.

Surgical reconstruction for these composite defects is quite difficult because multiple types of tissue from different anatomic sites are missing^1,2^. Reconstruction of these complicated defects not only requires restoring the anatomy but also replacing the exact amount and types of missing tissue. The application of free flaps to reconstruct composite oral and maxillofacial defects has been well reported in the literature^3–7^. However, few studies have discussed choosing different reconstructive solutions and designing free flaps based on a classification of composite oral and maxillofacial defects.

In the present study, we reviewed a series of patients with composite oral and maxillofacial defects and proposed a new classification method based on the elements and distribution of the missing tissues. Three types of reconstructive solutions using a free flap technique were developed accordingly. The reconstructive results using free flaps based on this classification were reported.

## Patients and Methods

### Patients

The clinical data of the patients diagnosed with oral and maxillofacial cancers from May 2009 to December 2016 in two tertiary cancer centre in southern China (Fujian Cancer Hospital and Sun Yat-sen University Cancer Center) were retrospectively reviewed. A total of 17 patients were included in this study. Among them, there were 6 females and 11 males, with a mean age of 49.4 years (range, 13 to 69 years). Pathologically, 13 patients were diagnosed with squamous cell carcinomas, 3 patients with sarcomas and one patient with adenoid cystic carcinoma (See Table 1). The treatment plan for all the patients was discussed with a multidisciplinary team. Postoperative radiotherapy was scheduled for most patients to improve local control. All patients received radical curative surgery and simultaneous reconstruction by free flaps. The composite oral and maxillofacial defects were evaluated and classified preoperatively by CT or MRI imaging. Reconstructive solutions using free flaps were planned individually according to different types of defects, the patients’ inclination and the surgeon’s preference for a specific surgical approach. All patients were followed up postoperatively in the clinic for at least two years.

**Table 1:** The clinical features of seventeen patients with composite oral and maxillofacial defects reconstructed using free flaps.

Case #	Sex	Age	Primary site	Pathology	Defect extent	Defects	Reconstruction	Flaps	Oral function
1	Female	64	Upper gingiva	SCC	Oral soft tissue + Facial skin	Type A	Perforator-based ALT	Survived	Good
2	Female	45	Cheek	SCC	Through-and-through cheek tissue	Type A	Perforator-based ALT	Survived	Good
3	Male	50	Floor of the mouth	SCC	Oral soft tissue + Facial skin	Type A	Perforator-based ALT	Survived	Good
4	Male	23	Facial and cervical skin	ACC	Wide-apart facial and cervical skin	Type B	Branch-based ALT	Survived	Good
5	Male	25	Mandible	Sarcoma	Oral soft tissue + Mandible	Type C	fibular flap	Survived	Common
6	Male	44	Lower gingiva	SCC	Oral soft tissue + Facial skin + Mandible	Type C	fibular flap	Survived	Common
7	Female	69	Floor of the mouth	SCC	Oral soft tissue + Facial skin	Type A	Perforator-based ALT	Survived	Good
8	Female	43	Lip	SCC	Lip + Cheek tissue	Type A	Perforator-based ALT	Survived	Good
9	Male	58	Hard palate	SCC	Oral soft tissue + Facial skin	Type A	Perforator-based ALT	Survived	Good
10	Female	55	Lower gingiva	SCC	Oral soft tissue + Mandible	Type C	fibular flap	Survived	Good
11	Male	64	Mandible	Sarcoma	Oral soft tissue + Mandible	Type C	fibular flap	Survived	Common
12	Male	56	Floor of the mouth	SCC	Oral soft tissue + Mandible	Type C	fibular flap	Survived	Good
13	Male	49	Floor of the mouth	SCC	Oral soft tissue + Mandible	Type C	fibular flap	Survived	Good
14	Male	69	Upper gingiva	SCC	Oral soft tissue + Facial skin	Type A	Perforator-based ALT	Survived	Good
15	Male	49	Lower gingiva	SCC	Oral soft tissue + Facial skin + Mandible	Type C	fibular flap	Survived	Good
16	Female	13	Upper jaw	Sarcoma	Oral soft tissue + Maxilla	Type C	fibular flap	Survived	Good
17	Male	64	Cheek	SCC	Through-and-through cheek tissue	Type A	Perforator-based ALT	Survived	Good

SCC, squamous cell carcinoma. ACC, adenoid cystic carcinoma.

The present study was approved by the institutional review board/ethics committee of the Cancer Center of SUN Yat-sen University and Cancer Hospital of FuJian Medical University. All methods were performed in accordance with the relevant guidelines and regulations. Written informed consent was obtained for all patients and/or their legal guardians (patients under the age of 18 years) in this study before surgery. Informed consent for both study participation and online open-access publication of identifying information and images was also obtained from all patients and/or their legal guardians (patients under the age of 18 years).

### Classification

All patients presented with composite oral and maxillofacial defects that included multiple types of tissues missed. We classified the composite defects according to two factors: the elements and distribution of the missing tissues. Elements referred to the five spatial structures of oral and maxillofacial regions that included the intra-oral mucosa, intermediate connective tissue, extra-oral facial skin, bony structures and the related cervical skin. Distribution referred to the spatial distance of the missing elements.

The elements of the missing tissue could be further classified into two types: soft tissue only and soft tissue combined with bone, including the mandible or maxilla. The distribution of the missing elements could be further classified into a close one-block pattern and a wide separate-block pattern.

Table 2 shows the four types of composite oral and maxillofacial defects according to this classification system. Both types A and B indicated soft tissue-only composite defects. The difference between types A and B was just the distance of the missing tissue elements. For example, type A defects lost some elements from the oral and maxillofacial regions in a close one-block pattern; however, type B defects lost tissue elements in a wide separate-block pattern. Both types C and D indicated composite defects with involvement of soft tissue and bone, including the mandible or maxilla. The difference between them also existed in a close one-block and wide separate-block pattern. The definitions for the types of composite oral and maxillofacial defects were as follows:

**Table 2:** Classification of the composite oral and maxillofacial defects.

	Distribution of the defects	
	Close one-block pattern	Wide separate-block pattern
**Tissue elements**		
Soft tissue only	Type A	Type B
Combined with bone	Type C	Type D
+ mandible		
+ maxilla		

Type A: Only elements of soft tissue missed in a close one-block pattern.

Type B: Only elements of soft tissue missed but in a wide separate-block pattern.

Type C: Both elements of soft tissue and bone missed in a close one-block pattern.

Type D: Both elements of soft tissue and bone missed but in a wide separate-block pattern.

### Reconstructive solutions

A composite oral and maxillofacial defect can be reconstructed in many ways. In the current work, free flaps with different designs were used to reconstruct these defects. Table 3 shows the general rule and our design of the free flaps to reconstruct different types of composite oral and maxillofacial defects. The anterolateral thigh flap and fibular flap were the main donor sites used to design the free flaps in this series. Three kinds of free flaps, including perforator-based ALT flaps, branch-based ALT flaps and well-designed fibular flaps, were developed for the reconstruction of the respective types of composite oral and maxillofacial defects.

**Table 3:** The reconstructive algorithm for different types of composite oral and maxillofacial defects.

Composite oral and maxillofacial defects	Reconstructive methods available	Designing of a free flap	Our solutions
Type A	(1) Double free flaps; (2) A folded free flap or local flap; (3) A well-designed free flap	Any free flaps designed to have two blocks of tissue based on two adjacent perforators	A perforator-based anterolateral thigh flap
Type B	(1) Double free flaps; (2) A well-designed free flap	Any free flaps designed to have two blocks of tissue based on two perforators separated widely apart	A branch-based anterolateral thigh flap
Type C	(1) Double free flaps; (2) A folded free flap or local flap −/+ bone reconstruction; (3) A well-designed free flap −/+ bone reconstruction	Any free flaps designed to have blocks of tissue including a segment of bone based on the adjacent perforators	(1) A fibular flap with or without chimeric design; (2) A perforator-based anterolateral thigh flap with or without a metal plate
Type D	(1) Double free flaps; (2) A folded free flap or local flap −/+ bone reconstruction; (3) A well-designed free flap −/+ bone reconstruction	Any free flaps designed to have blocks of tissue including segments of bone based on the perforators separated widely apart	No cases

Perforator-based ALT was defined as an ALT flap based on the perforators from the descending branch of the lateral circumflex femoral artery. These perforators were not distributed so far apart that the tissue blocks based on them had limited freedom of rotation.

Branch-based ALT was defined as an ALT flap based on the perforators coming from different branches of the lateral circumflex femoral artery, i.e., the descending branch and oblique branch. This design of the ALT flap was to obtain more freedom of rotation for wide-apart defects.

Fibular flaps could be designed individually according to the reconstructive requirement. Two methods are normally used, including one compound or chimeric style.

## Results

Among 17 patients, 8 patients presented with type A defects, one patient presented with a type B defect, and the other 8 patients presented with type C defects. No type D defects were observed in this series of patients (see Table 1). Perforator-based ALT flaps were used to reconstruct Type A defects. Branch-based ALT flaps were used to reconstruct Type B defects. For Type C defects, two reconstructive solutions were used, including a fibular flap and a perforator-based ALT flap with or without a metal plate for bony reconstruction. Representative cases for each type of defect reconstructed by a free flap are shown below.

All flaps survived after surgery. The patients were followed up in the clinic and reported no severe complications. All patients were able to resume an oral diet postoperatively. Fourteen patients reported good postoperative oral function, and three patients reported satisfactory postoperative oral function.

### Type A defects

**Figure 1:**
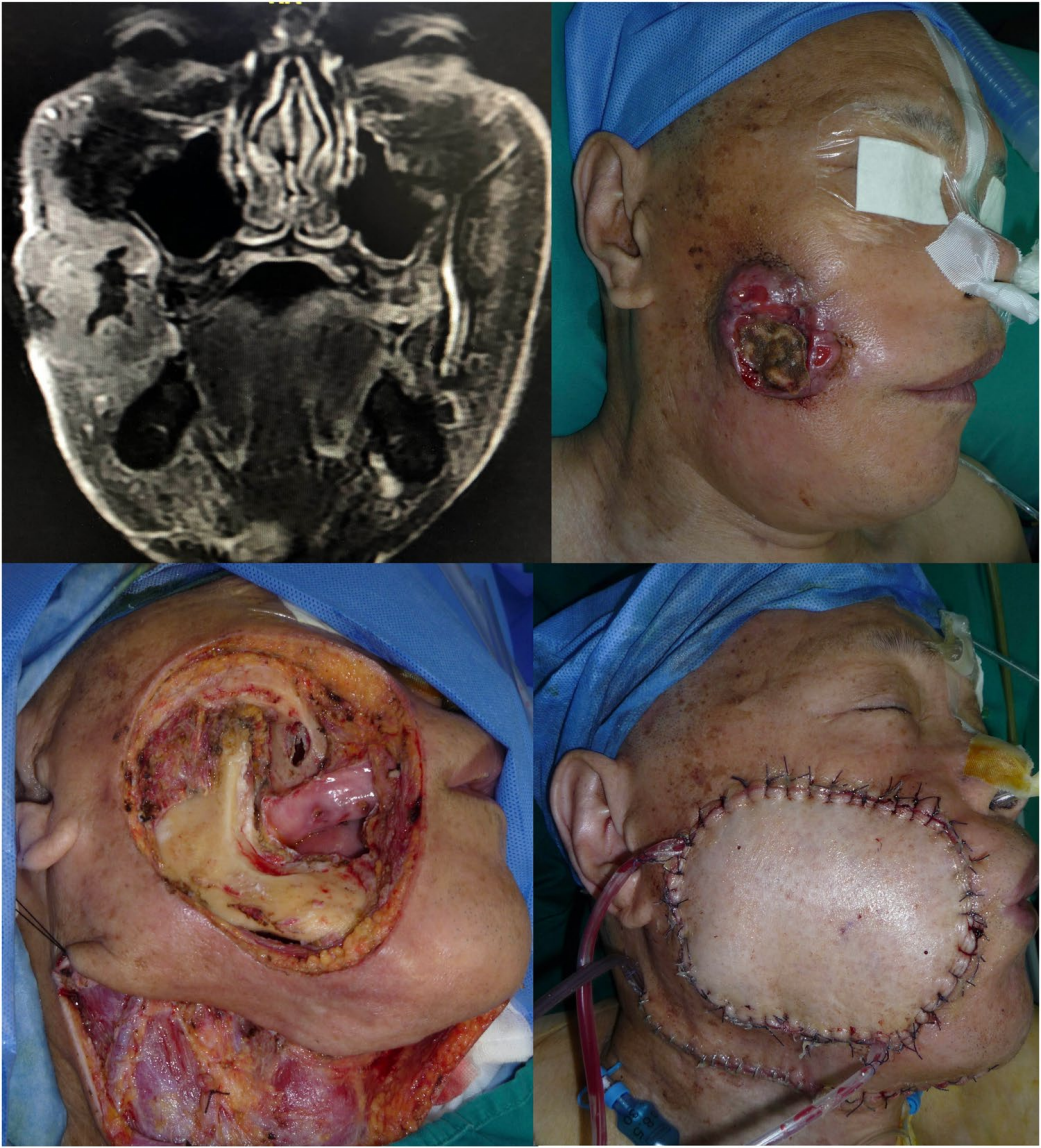
This patient presented with a one-block through-and-through oral soft tissue-only defect (type A) that was reconstructed by a perforator-based ALT flap.

**Figure 2:**
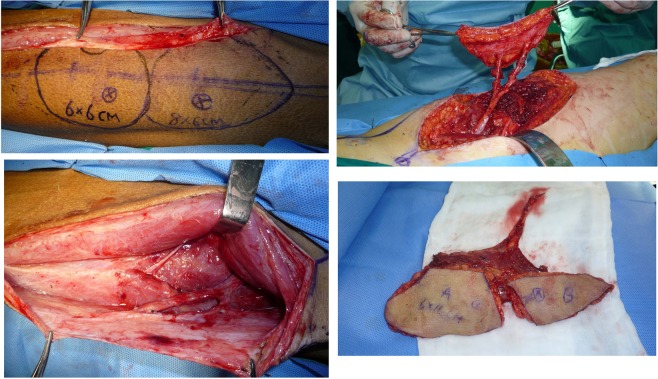
A perforator-based ALT flap was designed and harvested. Two blocks of donor tissue based on two adjacent perforators were prepared.

A 64-year-old gentleman presented with squamous cell carcinoma of the buccal mucosa. The tumour invaded the facial skin, but there was no evident bony invasion. A radical en bloc through-and-through resection was performed. The continuity of the mandible was maintained. This patient presented a Type A defect (only elements of soft tissue missed in a close one-block pattern). A perforator-based ALT flap was developed, and two blocks of tissue were prepared based on the two adjacent perforators. The Type A defect was well reconstructed by insetting the two blocks of tissue intra-orally and extra-orally to replace the missing mucosa and skin. (See Figs. 1 and 2.)

### Type B defects

**Figure 3:**
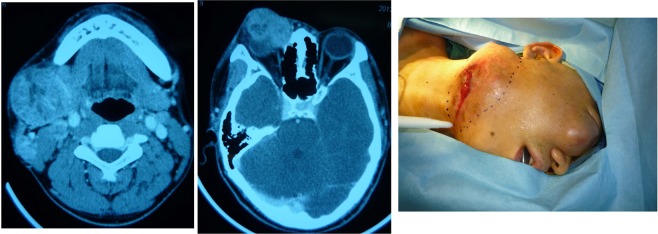
This patient presented with two blocks of soft tissue-only defects separated far apart (Type B) that was reconstructed by a branch-based ALT flap.

**Figure 4:**
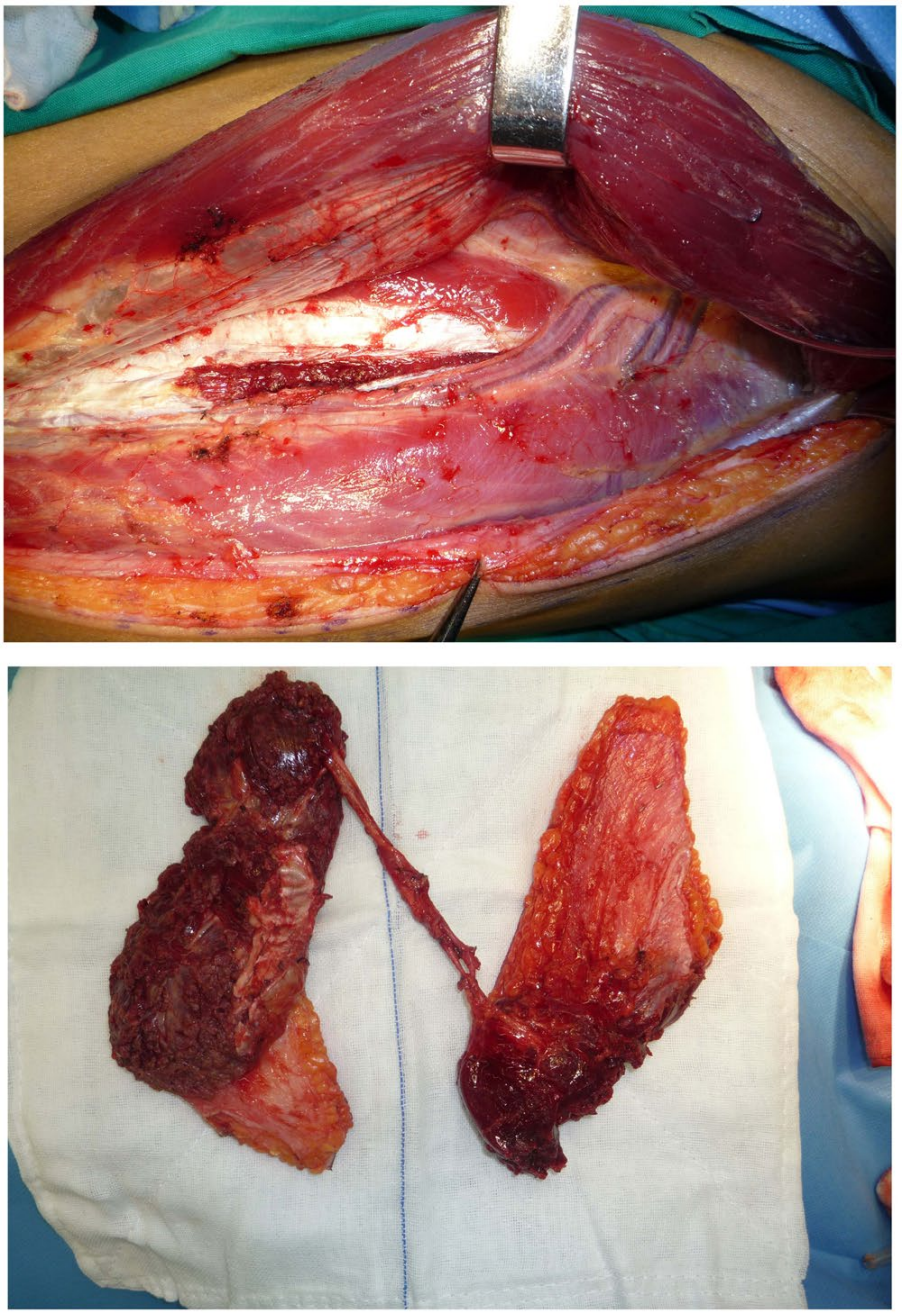
A branch-based ALT flap was designed and dissected based on the descending and oblique branches from the lateral circumflex femoral artery.

A 23-year-old gentleman presented with a mass located at the inner canthus with ipsilateral cervical swollen lymph nodes on level II. A biopsy pathologically confirmed an adenoid cystic carcinoma arising from the tarsal glands with cervical lymph node metastasis. CT scans showed that both the inner canthus and cervical skin were invaded. Radical surgery was performed to simultaneously remove the mass and neck nodes. The patient thus presented a Type B defect (only elements of soft tissue missed but in a separate-block pattern). Two branches, including the descending branch and the oblique branch of the lateral circumflex femoral artery, were identified and used to prepare two blocks of donor tissue, including the skin and muscle. A branch-based ALT flap was developed to reconstruct the two blocks of defects far apart from each other. (See Figs. 3 and 4.)

### Type C defects

#### Combined with the mandible

**Figure 5:**
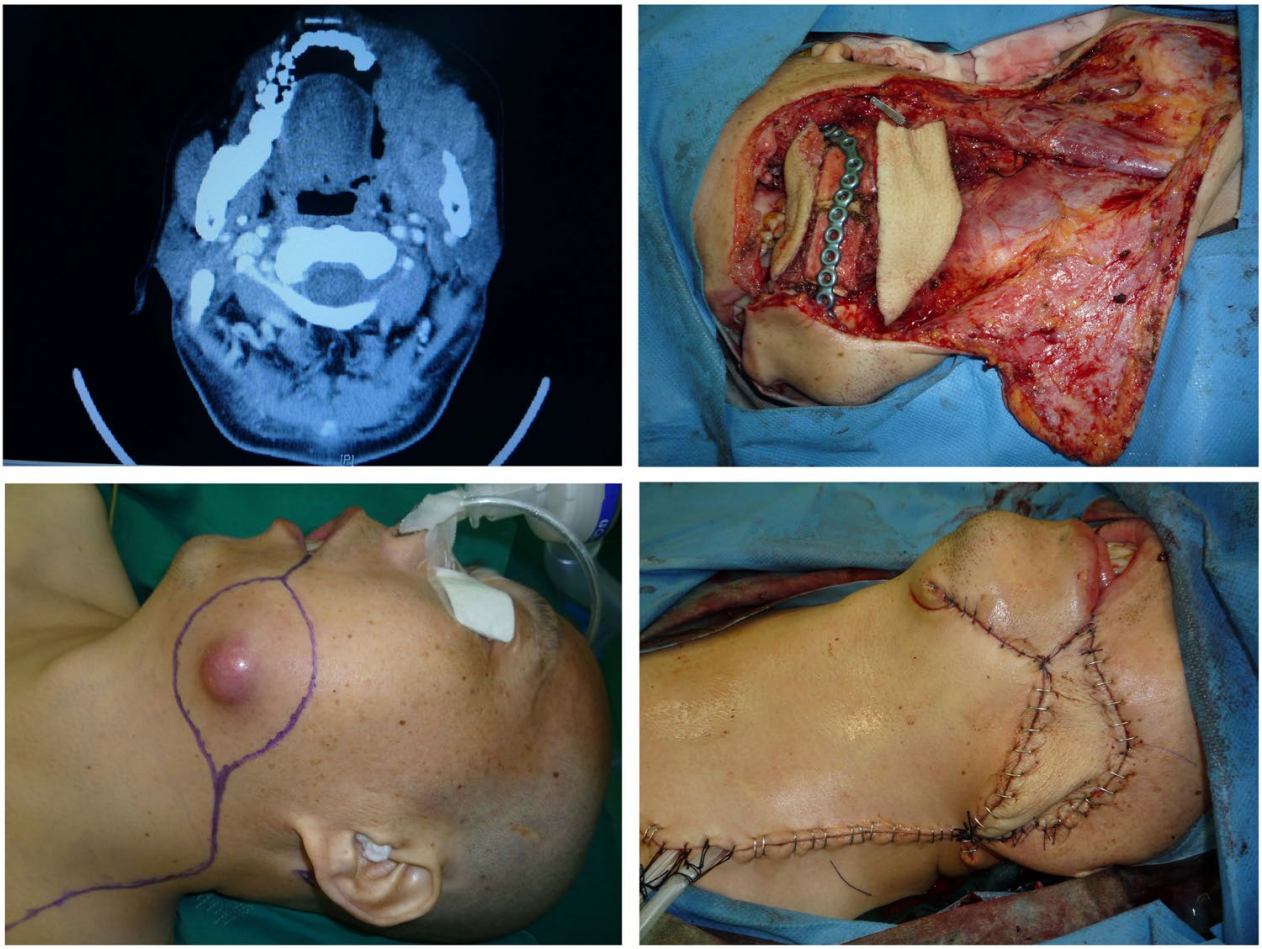
This patient presented with one-block through-and-through oral soft tissue combined with the mandible defect (Type C) that was reconstructed by a fibular flap with a chimeric design.

**Figure 6:**
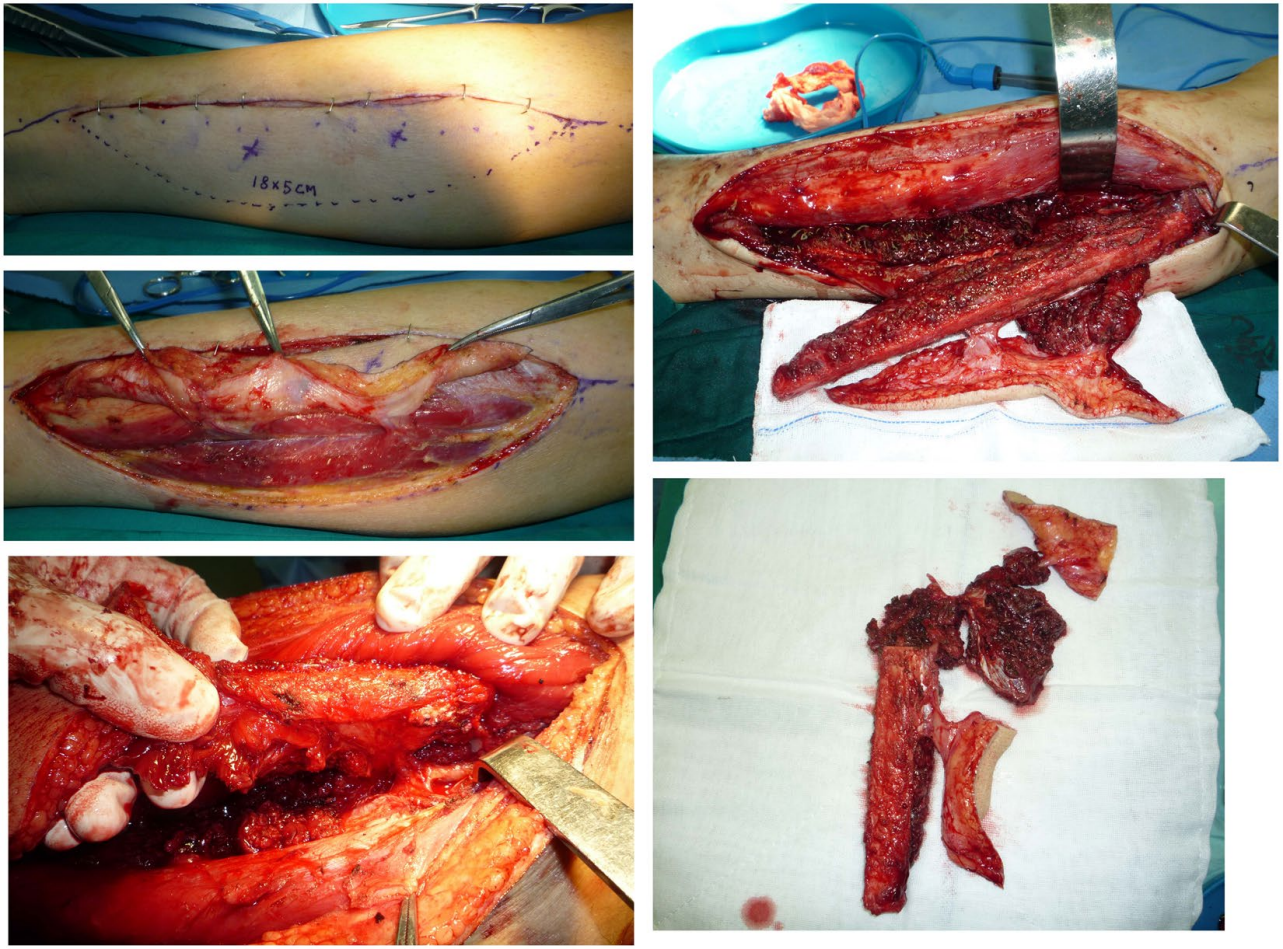
A fibular flap with a chimeric design with four blocks of tissue, including two skin paddles, one muscular block and a segment of bone, was dissected based on several perforators.

A 49-year-old gentleman presented with a T4N1M0 squamous cell carcinoma arising from the lower gingiva. A CT scan showed that the tumour had already invaded the body of the mandible. The extraoral facial skin was also invaded. Radical surgery was performed to remove a segment of the mandible with the whole three-layer of the cheek. This patient presented a Type C defect (both elements of soft tissue and bone missed in close one-block pattern). A fibular flap with chimeric design was then developed with two skin paddles, one block of the soleus muscle and one segment of the fibula to reconstruct the intra-oral mucosa, intermediate soft tissue and facial skin, respectively. (See Figs. 5 and 6.)

**Figure 7:**
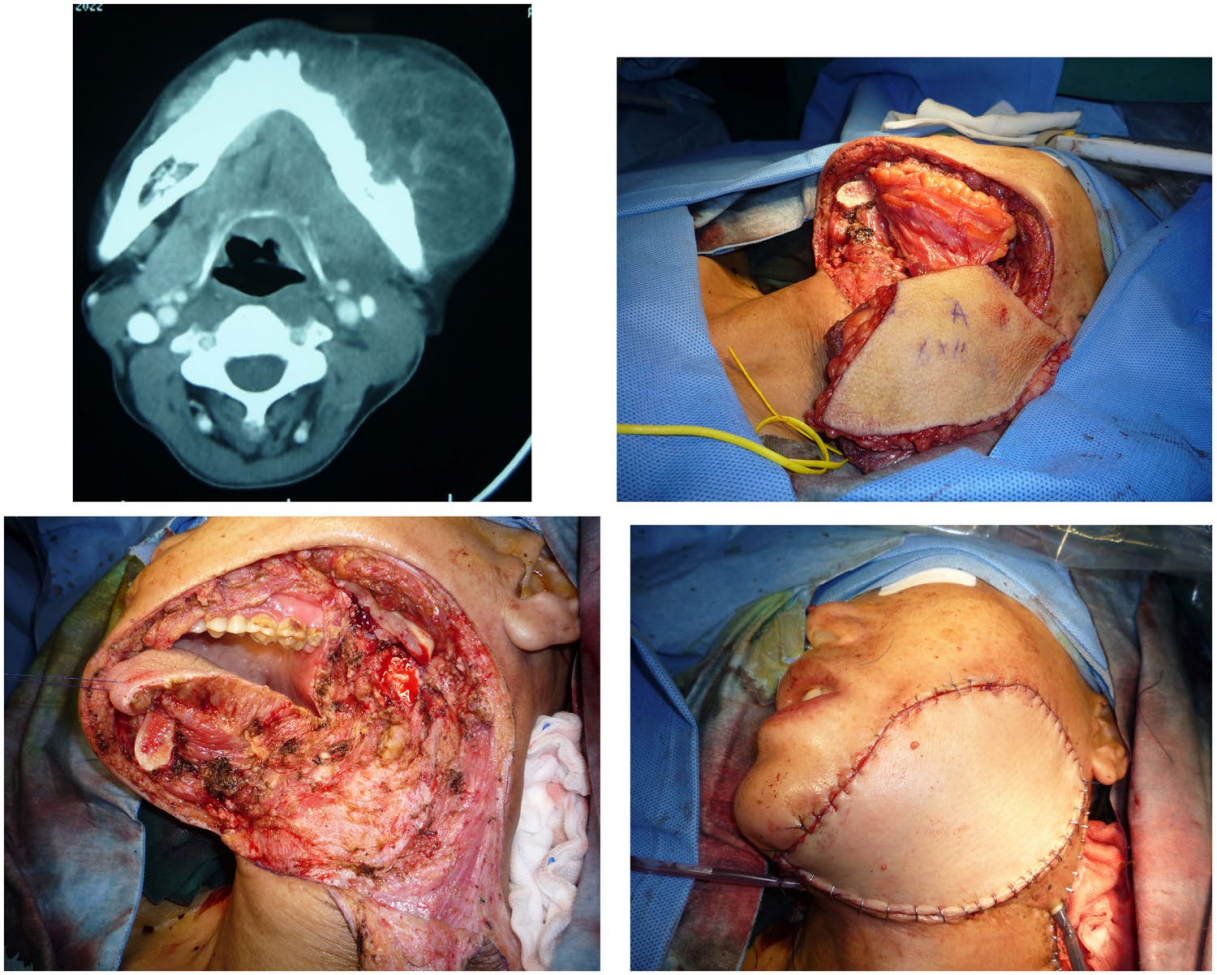
This patient with advanced cancer presented with a one-block through-and-through oral soft tissue combined with the mandible defect (Type C) after radical surgery. However, a perforator-based ALT flap was used to reconstruct only the soft tissue. The mandible was not reconstructed in consideration of the high potential of cancer recurrence.

Another 44-year-old male patient was confirmed pathologically to have a squamous cell carcinoma of the lower gingiva that had already invaded the mandibular bone. A Type C defect combined with the mandible was presented after radical ablative surgery. Because of the large mass with wide invasion, this Type C defect was repaired with a free ALT flap only for the soft tissue, without bone reconstruction. (See Fig. 7.)

#### Combined with the maxilla

**Figure 8:**
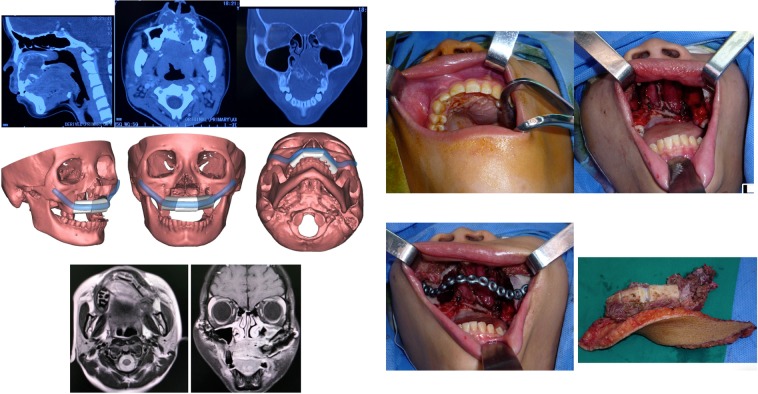
This patient presented with a one-block oral soft tissue combined with the maxilla defect (Type C) that was reconstructed by a fibular free flap plus a metal plate assisted by virtual surgical planning technology.

A 13-year-old girl presented with an extra-skeletal myxoid chondrosarcoma of the maxilla and received transoral radical surgery to remove the bilateral maxillary bone with preservation of a portion of the upper alveolar bone. This Type C defect combined with the maxilla was reconstructed by a one-compound free fibular flap designed by a virtual surgical planning technique. The missing upper alveolar ridge was replaced by a segment of fibular bone, and the intraoral soft tissue was replaced by a skin paddle. (See Fig. 8).

## Discussion

Composite defects are common after ablative surgery for the treatment of advanced oral and maxillofacial cancers. The key problem in this scenario is that different types and multiple layers of the missing tissues need to be reconstructed simultaneously. In addition, three-dimensional reconstruction requires donor tissue with a flexible folding capability and several islands of tissues. A well-designed free flap could provide flexible and multiple blocks of tissue to replace the missing tissue in these complicated defects. Understanding the features of the defects can guide the design and harvesting of the free flap. Clinical evaluation of composite oral and maxillofacial defects is an important step in the reconstruction of these defects.

The clinical evaluation of a defect determines the selection of a specific reconstructive technique^8^. In this study, we used two parameters (the elements and distribution of the missing tissues) to classify the composite oral and maxillofacial defects into four types. We believe this classification is an easy and applicable system, especially for helping to choose and design free flaps. The first parameter (elements) can indicate what types of tissue are needed, including skin, muscle or bone, and how many blocks of tissue should be prepared in the harvesting of a free flap. The second parameter (distribution) can help surgeons decide which perforators or branches should be based to harvest the free flap in order to obtain satisfactory insetting of different blocks of tissue without too much tension.

The current classification for composite oral and maxillofacial defects provided a basic framework to design a free flap in reconstruction. Many free flaps have been used in the reconstruction of head and neck defects, including anterolateral thigh flaps, rectus abdominal flaps, fibular flaps, scapular flaps, iliac spine flaps, etc. The choice of different flaps depends not only on the features of the defect but also on the surgeons’ familiarity with specific techniques. The general rules are that well-designed free flaps should provide different blocks of donor tissues based on suitable perforators regardless of which flaps are chosen. In this study, we performed all cases using free ALT and fibular flaps that had been the main reconstructive techniques in our practice.

The anterolateral thigh (ALT) flap is an optimal donor for reconstructing soft tissue-only defects (Type A/B) because it can provide enough tissue and can be designed in many ways. Successful preparation of a chimeric ALT flap requires identifying at least two sizable perforators and a common vessel trunk. Understanding the anatomical variation of the perforators is the most important factor to guarantee the application of the chimeric ALT flap. Previous studies have reported that an average of four musculocutaneous perforators could be identified per thigh despite the anatomical variations in ALT flaps. Moreover, only 7% of cases presented with only one perforator^9–11^. Therefore, the probability of harvesting a perforator-based ALT flap was very high. However, dissecting a branch-based ALT flap is not an easy task. Because the ascending and transversal branch of the lateral circumflex femoral artery (LCFA) is normally high and too deep to dissect, a branch-based chimeric ALT flap is often dissected based on the descending and oblique branch. Anatomical studies have reported the presence of an oblique branch in the ALT flap in only 34% of cases^9,12^; therefore, a branch-based chimeric ALT flap cannot be definitely dissected in a specific patient. A preoperative study of the anatomy of the LCFA using ultrasound or other imaging techniques should be performed to help design the flap^13,14^. Alternative surgical plans should be made preoperatively. Dissecting two separate free flaps and configuring them into one flow-through flap is one possible method in the concept of a chimeric flap. Using the vastus muscle (with or without a skin graft) as the second component of the ALT flap when a second skin paddle is not possible is another reasonable back-up plan in some situations. In addition, double free flaps or local flaps should also be prepared and used in patients with anatomical variations. Fortunately, a type B or D composite oral and maxillofacial defect is quite rare in clinical practice. In this study, we did not have patients with a type D defect, and only one patient presented with a type B defect. A branch-based chimeric ALT flap was used to reconstruct this rare type B composite defect, and its detailed technique was reported in our previous report^15^.

A type C defect with bone involvement is quite common after radical surgery for oral and maxillofacial cancers. The reconstruction of bone, including the mandible or the upper alveolar bone, is normally needed to obtain a satisfactory reconstructive result. However, for some advanced patients with a high potential risk of recurrent cancers, simple reconstruction without restoration of the bone or with a metal plate to replace the bone may be a better choice. Postoperative radiation can be performed as early as possible to increase the possibility of curing the cancer. In this study, we showed a case with a type C defect reconstructed only for the soft tissue using a perforator-based ALT flap without bony reconstruction, just like the procedure for a type A defect.

A well-designed fibular flap is an optimistic method for the reconstruction of a type C composite oral maxillofacial defect^16^. Anatomical studies have shown that cutaneous perforators can normally be identified along the posterior border in the middle or lower third of the fibula. The average number of cutaneous perforators was approximately 4 branches^17,18^. Our experiences demonstrated that designing two skin paddles was safe, and the flap could be stably dissected. Locating muscular perforators to the soleus was crucial for harvesting a chimeric fibular flap with blocks of muscle. A cadaveric study showed that two sizable muscular perforators to the soleus consistently branched from the proximal part of the peroneal artery^19,20^. After locating the main trunk of the peroneal artery, meticulous exploration should be performed to identify muscular branches to the posterior soleus muscle. In addition, a well-designed fibular flap is also a very good way to reconstruct a type C defect combined with the upper maxilla, as shown in this study. Virtual surgical planning could be used in this setting to achieve satisfactory positioning of the flap. Microvascular anastomosis of the short peroneal artery to the recipient vessel was another challenge in this case.

It should be noted that reconstruction of composite oral and maxillofacial defects using a free flap has several noteworthy limitations. This technique might not be applicable in some patients with anatomical variation of the perforator vessels, especially for the construction of branch-based ALT flaps. Identification of the perforators and harvesting of a free flap are tedious jobs. There is a long learning curve. To harvest a free flap may be very complicated and prone to failure because of the anatomical variation, especially when performed by an inexperienced surgeon. The number of cases using free flaps to reconstruct oral and maxillofacial defects in the current study is still very small. Much work is still needed in accumulating experience and developing practical methods to estimate the anatomical variation preoperatively in the future.

## Conclusions

The current classification system may help select and design specific free flaps to reconstruct composite oral and maxillofacial defects. Perforator-based, branch-based ALT flaps and well-designed fibular flaps could be successfully used to reconstruct the respective type A, B and C defects. The existence of anatomical variations in some patients should be noted before the application of this surgical technique.
